# Gendered patterns of common mental health outcomes and suicidal ideation after suicide bereavement and the role of perceived social support

**DOI:** 10.1093/heapro/daag122

**Published:** 2026-08-25

**Authors:** Eve Griffin, Grace Cully, Sophie Veronneau, Fiona Tuomey, Selena O’Connell, Karl Andriessen

**Affiliations:** National Suicide Research Foundation, 4.28 Western Gateway Building, Western Road, Cork T12 XF62, Ireland; School of Public Health, 4th Floor Western Gateway Building, Western Road, University College Cork, T12 XF62, Ireland; National Suicide Research Foundation, 4.28 Western Gateway Building, Western Road, Cork T12 XF62, Ireland; School of Medicine, Brookfield Health Science Complex, College Road, University College Cork, Cork T12 AK54, Ireland; HUGG, 13 Adelaide Road, Dublin D02 P950, Ireland; National Suicide Research Foundation, 4.28 Western Gateway Building, Western Road, Cork T12 XF62, Ireland; Centre for Mental Health and Community Wellbeing, Melbourne School of Population and Global Health, University of Melbourne, Grattan Street, Parkville, Melbourne, VIC 3010, Australia

**Keywords:** suicide bereavement, gender, postvention, mental health, social support

## Abstract

Each year ∼10 million people globally are bereaved by suicide. The aim of this study was to examine the gender-related impacts of suicide bereavement and the role of perceived social support. Data from a national Irish survey of adults bereaved or affected by suicide were used. In total, 2413 participants completed the survey. Both men and women reported poor levels of wellbeing and a high prevalence of moderate-severe depressive and anxiety symptoms, in addition to suicidal ideation. Men were significantly more likely to report current suicidal ideation (aIRR 1.57, 95% CI: 1.25–1.97), in addition to thoughts of self-harm (aIRR: 1.31, 95% CI: 1.09–1.57) or suicide (aIRR: 1.35, 95% CI: 1.13–1.60) related to the bereavement. High levels of perceived social support were associated with reduced risk of mental health and suicide-related outcomes, a pattern consistent for both men and women. The findings from this research have important implications for policy and practice, particularly in relation to screening for anxiety, depression, and suicidal ideation among those bereaved by suicide, in addition to the development and implementation of tailored postvention supports for men and women.

Contribution to Health Promotion

- Suicide is a significant public health issue, yet few studies have examined the broad gender-related impacts of suicide bereavement.
- Both men and women bereaved by suicide experience substantial mental health impacts as a result, and men are more at risk of thoughts of self-harm or suicide.
- Increased levels of perceived social support were found to be a protective factor for adverse outcomes for both men and women.
- These results have important implications for the development of tailored postvention supports, early identification of those at risk, and wider national suicide prevention policy.

## Introduction

About one in 20 people are exposed to a suicide death each year, and one in five experience such a loss during their lifetime (Andriessen *et al*. 2017). Research suggests that each suicide can affect between six immediate family members and 135 people in the community (Cerel *et al*. 2019). The impact of a death by suicide varies depending on the type and psychological closeness of the relationship, with strongest, often life-changing impacts mostly being reported after the suicide of a first-degree family member (Mitchell *et al*. 2009). Nonetheless, suicide is also likely to affect other family members, friends, colleagues, and acquaintances of the deceased person (Mitchell *et al*. 2009, Andriessen *et al*. 2017, Cerel *et al*. 2019).

Grief reactions that may occur after any cause of death include feelings of sadness, anger, and longing for the deceased. People bereaved by suicide may also experience more pronounced feelings of shock, shame, stigma, and rejection (Kõlves *et al*. 2019). They may feel angry toward themselves or the deceased person, struggle with understanding why the suicide had happened, and feel guilt for not being able to foresee and prevent the suicide (Jordan 2020).

Suicide bereavement is a risk factor for the onset of, or exacerbation of mental health problems, especially depression and posttraumatic stress disorder (PTSD; Erlangsen *et al*. 2017). People bereaved by suicide also have a three-fold risk of self-harm and suicide compared to those nonbereaved, and the risk is also elevated compared to those bereaved by other causes (Hill *et al*. 2020, Pitman *et al*. 2022, Alves *et al*. 2025). Evidence suggests that this increased risk may vary across genders, though few studies have specifically investigated such gender differences (Logan *et al*. 2024).

People bereaved by suicide may experience major challenges in their relationships after the bereavement, often resulting in diminished social support (Sajan *et al*. 2022), including experiencing or anticipating potentially negative reactions from others such as blame. Furthermore, some people in their social environment may lack skills to approach them (Azorina *et al*. 2019, Andriessen *et al*. 2020). A combination of emotional and social challenges may lead some suicide-bereaved people to withdraw socially or avoid conversations about their grief (Azorina *et al*. 2019, Andriessen *et al*. 2020, Sajan *et al*. 2022).

There are known gender differences in coping with grief, and risks for prolonged grief and mental health problems such as depression and PTSD (Stroebe *et al*. 2001, Maccallum *et al*. 2023). Cultural norms about coping could at least partly be responsible for these differences. For example, the traditional masculine ideal of self-reliance could enhance self-stigma and reduce help-seeking (Logan *et al*. 2024, Andriessen *et al*. 2025), a pattern seen in a recent Irish study where men were less likely than women to access formal types of bereavement support (Griffin *et al*. 2026). In some cases, psychosocial or somatic health complaints may lead to help-seeking from support groups or formal services (Kaspersen *et al*. 2022). However, help-seeking can be hampered by the perceived availability and expertise of services (Spillane *et al*. 2017). While the availability of social support has been shown to be an important factor in encouraging help-seeking (Griffin *et al*. 2026) and has been shown to be positively associated with posttraumatic growth (Levi-Belz *et al*. 2021, Whittaker *et al*. 2025), its relationship with mental health-related outcomes is less well understood.

The objective of the current study was to explore differences in self-reported impacts of suicide on mental health outcomes and suicidal ideation according to gender, and to explore the role of perceived social support on these outcomes.

## Methods

This manuscript was prepared in accordance with STROBE guidelines (von Elm *et al*. 2008).

### Study design and participants

The Irish Suicide Bereavement Survey was an online, cross-sectional survey which was open for participation between October 2021 and February 2022 (O’Connell *et al*. 2022). Participants were recruited in several ways, including via social media messaging and targeted advertisements, dissemination in print and digital media, and through organizations involved in supporting people bereaved by suicide. Particular efforts were made to recruit male participants and other groups who may be under-represented in research, including marginalized and ethnic minorities. This involved a particular call for participation for men via media channels and targeted recruitment via community sports groups and organizations representing men’s health. Individuals were eligible to participate if they were: aged 18 years and over, residing in Ireland, and identifying as being bereaved or affected by suicide. Where participants had experienced multiple bereavements, they were to consider the bereavement which had most impacted them.

### Data items

The survey contained both open and closed questions and consisted of four main sections: sociodemographic information, information about the death by suicide and its impact, current mental wellbeing of participants, and utilization of support services following bereavement. For the current study, only data captured via closed questions were utilized.

The sociodemographic information included: gender, age, ethnicity, area of residence, employment status, relationship status, religious beliefs, living arrangements, and sexual orientation. Survey participants were asked to describe their gender using the categories: male, female, and other (allowing participants to specify their identified gender using open text, e.g. nonbinary).

Details of the suicide bereavement included the number of bereavements, relationship to the deceased and time since bereavement.

### Outcome measures

*Mental wellbeing* was measured using the World Health Organization—Five Wellbeing Index (WHO-5; World Health Organization 1998). The WHO-5 is a self-report questionnaire, consisting of five statements relating to the previous 2 weeks. Raw scores range from 0 to 25 and are multiplied by four to get a total percentage score. A score of <50 is indicative of being clinically significant (World Health Organization 1998). The Cronbach’s Alpha score was α = 0.824.

*Anxiety and depression* symptoms were measured using the Patient Health Questionnaire—Anxiety and Depression Scale (PHQ-ADS; Kroenke *et al*. 2016). The PHQ-ADS incorporates DSM diagnostic criteria that is used for screening and monitoring depressive and anxiety symptoms. This measure consists of 16 statements and participants indicated how often they had been bothered by a problem over the past 2 weeks. Scores range from 0 to 48. Scores of ≥10, 20, or 30 are indicative of mild, moderate, and severe levels of depressive-anxiety symptomatology (Kroenke *et al*. 2016). The Cronbach’s Alpha score was α = 0.934.

The extent to which participants expressed thoughts of self-harm or suicide over the past 2 weeks (referred to in this study as *current suicidal ideation*) was measured using item 9 of the PHQ-ADS (‘How often have you been bothered by thoughts that you would be better off dead or of hurting yourself in some way?’). We created a binary variable, with responses of ‘several days’, ‘more than half the days’, or ‘nearly every day’ indicative of suicidal and/or self-harm ideation.

We also measured *suicidal ideation as a result of the bereavement*. Two items of the Grief Experience Questionnaire (GEQ; Bailley *et al*. 2000) were used to indicate self-harm ideation (‘Worry that you might harm yourself since the death’) and suicide-related ideation (‘Think of ending your own life since the death’). We created binary variables, with responses of ‘sometimes’, ‘often’ or ‘almost always’ indicative of self-harm or suicide-related ideation as a result of bereavement.

*Perceived impact* of the bereavement was measured via one item, with response options ranging from 0 (no impact) to 3 (major impact). The responses were dichotomized for the current study (no/little impact and moderate/major impact). In addition, participants were asked to indicate whether their bereavement had impacted on different aspects of their health and life, using a question adapted from McDonnell *et al.* (2020).

### Statistical analysis

Descriptive statistics were used to examine the characteristics of participants. Proportions are reported for categorical variables and the mean and standard deviation for continuous variables. Differences in categorical variables between groups were examined using chi-square tests. Gender differences in all outcome measures were assessed using Poisson regression models which are more appropriate than Logistic regression for common outcomes (Aljawadi 2025), with incidence rate ratios (IRRs) and their 95% confidence intervals (95% CIs) reported. Robust standard errors were used. Variables associated with gender (*P* < 0.05) were entered into the models as covariates. Models which examined age-specific differences and impacts of social support were stratified by gender. The multivariate Poisson regression had 95% power to detect a gender difference represented by an IRR of at least 1.2. All analyses were conducted using Stata/BE Version 17.0.

### Ethical approval

The study received full ethical approval from the Clinical Research Ethics Committee of the Cork Teaching Hospitals [ECM 4 (j)10/8/2021 & ECM 3 (rr) 07/09/2021].

## Results

In total, 2413 participants completed the survey. Of these, gender was not recorded for 8 (0.3%) responses. Most participants were women (*n* = 1752; 73.1%), 644 were men (26.9%) and a minority identified as another gender (*n* = 9; 0.4%). Due to the small number in the latter category, our analyses focus on the gender categories of men and women, representing a total sample of 2396 (99.3%).

The sociodemographic profile of survey participants is outlined in Table 1. There were no significant differences between men and women in terms of ethnicity, employment status, relationship status, or living arrangements. However, men were more like to be of older age (22.0% vs. 16.9% aged 55+ years; χ^2^[1] = 16.40, *P* < 0.001); to identify as nonreligious (40.3% vs. 27.1%; χ^2^[1] = 29.64, *P* < 0.001); and were less likely than women to identify as heterosexual (88.9% vs. 94.6%; χ^2^[1] = 18.87, *P* < 0.001).

**Table 1:** Sociodemographic and bereavement characteristics of sample (*n* = 2396).

	Men (*n* = 644; 26.9%)	Women *n* = 1752 (73.1%)	Chi-square (degrees of freedom); *P*-value
Age group^a^			
18–24 years	28 (4.4%)	122 (7.0%)	16.40 (3); *P* < 0.001
25–34 years	127 (19.8%)	294 (16.9%)	
35–54 years	344 (53.8)	1026 (59.1)	
55+ years	141 (22.0%)	294 (16.9%)	
Ethnicity^b^			
White Irish	495 (95.6%)	1387 (95.6%)	0.01 (1); 0.978
Other	23 (4.4%)	64 (4.4%)	
Religion^c^			
Religious	289 (59.7%)	1006 (72.9%)	29.64 (1); <0.001
Nonreligious	195 (40.3%)	373 (27.1%)	
Sexual orientation^d^			
Heterosexual	455 (88.9%)	1354 (94.6%)	
Other	57 (11.1%)	78 (5.5%)	18.87 (1); <0.001
Relationship status^e^			2.42 (2); 0.299
In a relationship	380 (73.5%)	1014 (70.4%)	
Single	92 (17.8%)	302 (20.9%)	
Separated/divorced/widowed	45 (8.7%)	125 (8.7%)	
Experienced multiple bereavements	364 (56.5%)	929 (53.0%)	2.32 (1); 0.128
Relationship to deceased^f^			
Family member/Partner	357 (56.6%)	1230 (71.2%)	47.55 (2); 0.001
Friend/acquaintance	245 (38.8%)	426 (24.7%)	
Bereaved in a professional capacity	29 (4.6%)	71 (4.1%)	
Time since bereavement			
10 years or less	425 (66.0)	1219 (69.6%)	2.81 (1); 0.094
More than 10 years	219 (34.0)	533 (30.4)	
Perceived social support (mean, SD)^g^	14.1 (5.01)	14.8 (4.56)	T = −3.32(2370); <0.001

^a^Age not known for 20 participants. ^b^Ethnicity not known for 427 participants. ^c^Religiosity not known for 533 participants. ^d^Sexual orientation not known for 452 participants. ^e^Relationship status not known for 438 participants. ^f^Relationship to the deceased not known for 35 participants. ^g^Degree of perceived social support not known for 24 participants.

The number of suicide bereavements reported and the time since bereavement did not differ between men and women (Table 1). However, there were significant differences in the reported relationship to the deceased. Women were more likely to have experienced the loss of a family member/partner (71.2% vs. 56.5%; χ^2^[2] = 47.55, *P* < 0.001), whereas men were more like to report the loss of a friend/acquaintance (38.8% vs. 24.7%). Women were more likely to report higher levels of perceived social support than men (Mean score 14.8 vs. 14.1; *t* = 3.32[2370], *P* < 0.001).

### Impacts of the bereavement

There was no significant difference between men and women in the number of impacts reported as a result of the bereavement (see Table 2). Overall, women were more likely to rate the impact of the suicide death as moderate/major, compared to men (93.9% vs. 88.5%; χ^2^[1] = 19.64, *P* < 0.001). There were also differences in the types of impacts experienced. Women were more likely to report relational impacts (40.6% vs. 31.7%; χ^2^[1] = 16.01, *P* < 0.001), whereas men were more likely to report addictive behaviors, specifically prolonged use of alcohol and illegal/prescription drugs (for more than 3 months) (29.9% vs. 18.9%; χ^2^[1] = 34.61, *P* < 0.001). Almost half of participants reported mental or physical health impacts as a result of the bereavement, which was similar for men and women (46.7% and 49.7%, respectively).

**Table 2:** Reported impacts of the bereavement according to gender.

	Men (*n* = 644; 26.9%)	Women (*n* = 1752; 73.1%)	Chi-square (degrees of freedom); *P*-value
Number of adverse impacts reported			
One	130 (32.2%)	394 (33.9%)	0.67(2); 0.716
Two	86 (21.3%)	254 (21.9%)	
Three or more	188 (46.5%)	514 (44.2%)	
Bereavement impact			
No/minor	74 (11.5%)	107 (6.1%)	19.642 (1); <0.001
Moderate/major	569 (88.5%)	1645 (93.9%)	
Type of impact			
Relational impacts	204 (31.7%)	712 (40.6%)	16.012 (1); <0.001
Financial/employment	84 (13.0%)	210 (11.9%)	0.49 (1); 0.484
Living arrangements	53 (8.2%)	152 (8.7%)	0.12 (1); 0.729
Addictive behaviors	193 (29.9%)	329 (18.8%)	34.61 (1); <0.001
Mental or physical health	301 (46.7%)	870 (49.7%)	1.61 (1); 0.205

### Mental health and suicide-related outcomes

The proportion of participants reporting poor levels of current wellbeing (at the time of completing the survey, represented by a WHO-5 score of ≤50) was similar for men and women (52.6% and 56.6%, respectively; see Table 3). In addition, the proportion of participants reporting moderate-to-severe depressive and anxiety symptoms was similar for both genders (depressive symptoms: 32.5% and 30.9% respectively; anxiety symptoms: 26.1% and 26.6%, respectively).

**Table 3:** Differences in mental health and suicide-related outcomes according to gender.

	Male *N* (%)	Female *N* (%)	IRR (95% CI)	*P*-value	aIRR^a^ (95% CI)	*P*-value
Wellbeing (WHO-5 score ≤50)	328 (52.6)	971 (56.6)	0.93 (0.82–1.05)	0.252	0.93 (0.80–1.08)	0.369
Moderate-severe depressive symptoms (PHQ-9 Score ≥10)	207 (32.5)	537 (30.9)	1.04 (0.89–1.23)	0.552	0.99 (0.82–1.22)	0.995
Moderate-severe anxiety symptoms (GAD Score ≥10)	163 (26.1)	450 (26.6)	0.98 (0.82–1.17)	0.840	0.95 (0.76–1.18)	0.648
Thoughts of self-harm or suicide in the past 2 weeks (PHQ-9)	174 (27.4)	312 (18.0)	1.52 (1.26–1.83)	<0.001	**1.57** (**1.25–1.97)**	**<0**.**001**
Thoughts of self-harm following the bereavement (GEQ)	249 (41.1)	525 (32.3)	1.27 (1.09–1.46)	0.002	**1.31** (**1.09–1.57)**	**0**.**003**
Thoughts of suicide following the bereavement (GEQ)	266 (43.8)	539 (32.1)	1.37 (1.18)	<0.001	**1.35** (**1.13–1.60)**	**0**.**001**

Significant associations (*P* < 0.05) are highlighted in bold. aIRR, adjusted IRR.

^a^Adjusted for age, religiosity, sexual orientation, relationship to deceased and bereavement impact, perceived social support.

Men were more likely to report experiencing thoughts of self-harm or suicide than women. More than one-quarter of male participants (27.4%) reported some thoughts of self-harm or suicide in the 2 weeks prior to completing the survey, compared to 18.0% of women (aIRR 1.57; 95% CI 1.25–1.97). Men were also more likely to report thoughts of self-harm or suicide as a direct result of their bereavement. In total, 41.1% of men reported thoughts of harming themselves following the death, compared to 32.3% of women (aIRR 1.31, 95% CI 1.09–1.57), while 43.8% of men reported thoughts of ending their life, compared to 32.1% of women (aIRR 1.35, 95% CI 1.13–1.60).

In terms of age differences, younger adults (those aged 18–34 years) were more likely to experience poor current wellbeing and moderate-severe symptoms of depression and anxiety than older participants (see Supplementary Table S1). Strikingly, the proportion of participants who reported suicidal ideation was similar for young men and women. There were no differences between men and women aged 18–34 years in current suicidal ideation (32.5% and 30.7%, respectively; aIRR 1.07, 95% CI 0.72–1.59), thoughts of harming themselves following the death (43.4% and 44.6%, respectively; aIRR 0.97, 95% CI 0.69–1.37) or thoughts of ending of their life following the death (44.8% and 42.5%, respectively; aIRR 1.02, 95% CI 0.72–1.44). The suicide-related variables decreased with age for women, but not for men.

### Association between perceived social support and mental health and suicide-related outcomes

Reporting high levels of perceived social support was associated with reduced risk of all mental health and suicide-related outcomes (see Table 4). This pattern was consistent for both men and women, with aIRRs ranging from 0.34 to 0.52 for men and 0.27 to 0.61 for women.

**Table 4:** Association between perceived social support and mental health and suicide-related outcomes, according to gender.

	Male				Female			
	Low social support (*N*, %)	High social support (*N*, %)	aIRR^a^ (95% CI)	*P*-value	Low social support (*N*, %)	High social support (*N*, %)	aIRR^a^ (95% CI)	*P*-value
Wellbeing (WHO-5 score ≤50)	254 (65.9)	74 (30.9)	**0.48** (**0.35–0.65)**	**<0**.**001**	671 (69.3)	300 (40.1)	**0.61** (**0.52–0.71)**	**<0**.**001**
Moderate-severe depressive symptoms (PHQ-9 Score ≥10)	167 (42.4)	40 (16.5)	**0.42** (**0.28–0.63)**	**<0**.**001**	406 (41.5)	131 (17.3)	**0.43** (**0.34–0.53)**	**<0**.**001**
Moderate-severe anxiety symptoms (GAD Score ≥10)	130 (33.5)	33 (13.9)	**0.42** (**0.27–0.65)**	**<0**.**001**	329 (34.5)	121 (16.4)	**0.54** (**0.42–0.68)**	**<0**.**001**
Thoughts of self-harm or suicide in the past 2 weeks (PHQ-9)	146 (37.1)	28 (11.6)	**0.34** (**0.21–0.54)**	**<0**.**001**	251 (25.7)	61 (8.1)	**0.27** (**0.19–0.39)**	**<0**.**001**
Thoughts of self-harm following the bereavement (GEQ)	189 (50.4)	60 (25.9)	**0.52** (**0.37–0.72)**	**<0**.**001**	378 (39.9)	167 (22.7)	**0.59** (**0.48–0.73)**	**<0**.**001**
Thoughts of suicide following the bereavement (GEQ)	210 (55.9)	26 (24.2)	**0.45** (**0.32–0.63)**	**<0**.**001**	385 (40.7)	154 (20.9)	**0.51** (**0.41–0.63)**	**<0**.**001**

Significant associations (*P* < 0.005) are highlighted in bold. aIRR, adjusted IRR.

^a^Adjusted for age, religiosity, sexual orientation, relationship to deceased and bereavement impact.

## Discussion

### Main finding of this study

This is one of the few studies to explore the impacts of suicide bereavement according to men and women. The findings of this research underscore the profound impacts on both men and women, both mental and physical, regardless of the relationship to the deceased, particularly among young people. There were differences in the types of impacts reported by men and women, with women more likely to report family and relationship impacts, while men were more likely to report legal issues and prolonged substance use. Furthermore, compared to bereaved women, there was an increased risk of suicidality observed among bereaved men.

### What is already known on this topic

Few studies have comprehensively examined gender differences with regards wide-ranging mental health impacts of suicide bereavement. Previous studies using this same data source have highlighted that men are less likely to access supports following suicide bereavement (Griffin *et al*. 2026). A large-scale Danish study found that suicide bereavement conferred an increased risk of suicide compared to other types of bereavement, regardless of sex (Pitman *et al*. 2022), while a recent systematic review reported that gender-specific factors, including cultural norms and coping strategies, significantly influence men’s experiences of suicide bereavement (Logan *et al*. 2024).

### What this study adds

Our findings indicate that suicide bereavement has profound impacts on both men and women, regardless of their relationship to the deceased. Bereaved individuals reported poor quality of life, along with high levels of anxiety, depression, and suicidality. Overall, the proportion of our bereaved sample reporting poor wellbeing was higher than that reported in the general population (41%; Domenech *et al*. 2025). The prevalence of mild-moderate anxiety and depressive symptoms were also much higher for both men and women compared with the general population (Troya *et al*. 2022). In the general population, these symptoms are typically more common in women than in men (Somers *et al*. 2006, Salk *et al*. 2017). However, in our study, prevalence rates were similar for bereaved men and women. This contrasts with general population patterns and indicate that suicide-bereaved men may experience a greater relative increase in mental health symptoms compared with suicide-bereaved women.

Men were 1.3 times more likely to report thoughts of suicide and self-harm following the bereavement. This is significant, particularly as women are more likely to experience a suicide bereavement (Pitman *et al*. 2023). Recent qualitative research with suicide-bereaved men also revealed how participants struggled with suicidal ideation or behavior as a result of their bereavement (Andriessen *et al*. 2025). While previous research has found than men bereaved by suicide are at increased risk of suicide mortality, the existing evidence around suicidal ideation is more mixed (Logan *et al*. 2024). Strikingly, this higher rate of suicidal ideation held when adjusting for relationship to the deceased. In our study, more than one-third of men reported the loss of a work colleague or friend, which reinforces the adverse impacts of a suicide among unrelated individuals. While we did not measure it directly in the current study, these findings further emphasize the importance of considering degree of perceived closeness to the deceased in the context of providing support following a suicide (Brown *et al*. 2024). In addition to the importance of providing support to family members bereaved by suicide, the development of supports and intervention approaches for men who have lost a friend, classmate, or work colleague to suicide is warranted, especially as men in our sample were more likely to have been bereaved by a suicide of a nonfamily member than women.

These patterns were evident among young adults also, for both men and women. Approximately one-third had current thoughts of suicide or self-harm at the time of completing the survey, and a higher proportion, ∼40%, had thoughts of self-harm or suicide as a result of the bereavement. The potential contagion effect of suicide among young people—a UK study found that one in 11 young people who died by suicide were themselves bereaved by suicide (Rodway *et al*. 2022)—highlights the need for coordinated supports to support young people following a death by suicide, particularly within educational and community settings (Allie *et al*. 2023, De Oliveira *et al*. 2023).

It is well established that men are less likely to seek help for mental health conditions more generally (Lynch *et al*. 2018), in addition to accessing postvention support following a death by suicide (Pitman *et al*. 2017, McDonnell *et al*. 2022, Griffin *et al*. 2026). The observed gender differences in this study in terms of the magnitude and types of impacts reported are likely to be reflections of cultural norms around grieving and masculine ideals of self-reliance, which can in turn lead to increased suicidality (King *et al*. 2020). Such internalization can lead to additional maladaptive coping strategies such as substance misuse, reflected also in our current findings. In this study, the degree of perceived social support has emerged as an important protective factor: high levels of social support among both men and women were associated with better overall wellbeing, lower prevalence of depressive and anxiety symptoms and thoughts of self-harm and suicide. Social support following a bereavement has long been acknowledged as an important component of postvention (Hofmann *et al*. 2025). In particular, practical and emotional support in the immediate aftermath of a suicide have been highly valued among those bereaved (Ross *et al*. 2021, O’Connell *et al*. 2022). This also mirrors findings from a previous study using this sample where perceived social support is a strong factor in people accessing postvention supports and services (Griffin *et al*. 2026), and where informal supports—including those from family and friends—were associated with positive personal growth for men (Griffin *et al*. 2026). Therefore, the current findings provide strong evidence to develop approaches which seek to enhance the quality of social support for people bereaved by suicide, and men in particular. This could be addressed through educational initiatives for the general population on grief literacy and how best to support and signpost people to evidence-based services.

The findings from this research have important implications for policy and practice, particularly in relation to the importance of screening for symptoms of anxiety, depression, and suicidal ideation among those bereaved by suicide, in addition to the development and implementation of tailored postvention supports for men and women.

### Limitations of this study

We used data from a comprehensive national survey. The large sample size allowed for gender comparisons. The lower response rate from men (27%) is still higher than might be expected in similar suicide and health-related research. While we used a broad and inclusive definition of gender, we did not have sufficient data to analyze responses from participants who identified as a gender other than male or female. Future research should consider recruitment strategies to examine impacts across different gender categories. The cross-sectional survey design meant we were not able to establish a causal association between gender, perceived social support and the mental health-related outcomes examined. We also were not able to determine levels of mental health outcomes prior to suicide bereavement. Nevertheless, we were able to explore clear associations in terms of suicidal ideation experienced as a direct result of the bereavement. We also used a well-established measure of current suicidal ideation (PHQ-9). However due to the phrasing of this item, it was not possible to distinguish prevalence of self-harm and suicidal ideation separately.

## Supplementary Material

daag122_Supplementary_Data

## Data Availability

To access the AfterWords survey data, please contact the corresponding author.
