# Supplementary material for: Gendered patterns of common mental health outcomes and suicidal ideation after suicide bereavement and the role of perceived social support
Source: Health Promot Int. 2026 Aug 25;41(4):daag122. doi: 10.1093/heapro/daag122 (PMC13504280; doi:10.1093/heapro/daag122)
Supplement: Supplementary file 1 [file daag122_supplementary_data.docx]

**Supplementary Table 1. Differences in mental health and suicide-related outcomes according to gender and age group**

|  | **18-34 years** | | | | **35-54 years** | | | | **55+ years** | | | |
| --- | --- | --- | --- | --- | --- | --- | --- | --- | --- | --- | --- | --- |
|  | **Male**  **N (%)** | **Female**  **N (%)** | **aIRR (95% CI)** | **p-value** | **Male**  **N (%)** | **Female**  **N (%)** | **aIRR (95% CI)** | **p-value** | **Male**  **N (%)** | **Female**  **N (%)** | **aIRR (95% CI)** | **p-value** |
| Wellbeing (WHO-5 score ≤50) | 86 (55.8) | 274 (66.0) | 0.87 (0.65-1.16) | 0.346 | 172 (51.8) | 567 (56.1) | 0.91 (0.75-1.12) | 0.376 | 67 (50.8) | 125 (45.1) | 1.20 (0.83-1.71) | 0.329 |
| Moderate-severe depressive symptoms (PHQ-9 Score ≥10) | 63 (40.6) | 193 (46.5) | 0.92 (0.65-1.30) | 0.631 | 107 (31.6) | 263 (25.7) | 1.09 (0.82-1.43) | 0.555 | 37 (26.6) | 79 (27.9) | 1.20 (0.83-1.71) | 0.329 |
| Moderate-severe anxiety symptoms (GAD Score ≥10) | 55 (33.9) | 180 (43.7) | 0.79 (0.55-1.15) | 0.232 | 87 (26.1) | 223 (22.3) | 1.07 (0.79-1.44) | 0.668 | 24 (17.6) | 46 (16.9) | 0.91 (0.48-1.73) | 0.783 |
| Thoughts of self-harm or suicide in the past two weeks (PHQ-9) | 50 (32.5) | 127 (30.7) | 1.07 (0.72-1.59) | 0.742 | 86 (25.4) | 143 (14.0) | **1.95 (1.41-2.69)** | **<0.001** | 37 (26.6) | 40 (14.1) | **1.80 (1.04-3.11)** | **0.034** |
| Thoughts of self-harm following the bereavement (GEQ) | 62 (43.4) | 178 (44.6) | 0.97 (0.69-1.37) | 0.866 | 131 (39.8) | 290 (29.3) | **1.45 (1.14-1.85)** | **0.003** | 54 (41.5) | 70 (24.8) | 1.49 (0.98-2.28) | 0.062 |
| Thoughts of suicide following the bereavement (GEQ) | 64 (44.8) | 169 (42.5) | 1.02 (0.72-1.44) | 0.900 | 142 (43.2) | 292 (29.5) | **1.48 (1.17-1.88)** | **<0.001** | **58 (44.3)** | **71 (25.5)** | **1.52 (1.01-2.29)** | **0.044** |

aIRR=Adjusted IRR.

*Adjusted for age, religiosity, sexual orientation, relationship to deceased and bereavement impact, perceived social support
